# In Honor of Dr. Roswell Park

**Published:** 1891-06

**Authors:** 


### IN HONOR OF DR. ROSWELL PARK.

In the May number of this Journal we published an obituary of Dr. Chas. T. Parkes, late Professor of Surgery in Rush Medical College, Chicago, Ill. It appears that soon after the chair became vacant through this sad event, the Faculty decided to divide it into two departments, tendering one to Dr. N. Senn, and the other to Dr. Roswell Park of this city. Dr. Park, after due consideration, declined to accept, hence will remain in Buffalo. His many friends, in and out of the profession, thereupon united in tendering him a banquet in appreciation of his decision. The date fixed upon therefor was May 18th, but the sudden death of Dr. Burwell, who was one of the principal movers in the project, caused a postponement to May 25, 1891, and it is now being held even while these lines are printing. We hope to speak further of the event in our next issue.

---

The Buffalo Woman's Hospital is now permanently established at No. 191 Georgia street, corner Seventh. The hospital is a growth of the work previously carried on at the Maternity Hospital, and aims now to enlarge its usefulness to the public and the profession, by treating all diseases peculiar to women. The character and ability of the attending and consulting staff is a guaranty of the excellent work this institution will do. We commend it to the profession—from which it merits a liberal patronage.

